# Cone‐beam computed tomography analysis of lingual mandibular bone depression in the premolar region: A case report

**DOI:** 10.1002/ccr3.2713

**Published:** 2020-02-11

**Authors:** Saeed Asgary, Naghmeh Emadi

**Affiliations:** ^1^ Iranian Center for Endodontic Research Research Institute of Dental Sciences Shahid Beheshti University of Medical Sciences Tehran Iran; ^2^ Dentofacial Deformity Research Center Research Institute of Dental Sciences Shahid Beheshti University of Medical Sciences Tehran Iran

**Keywords:** cone‐beam computed tomography, density, mandibular bone depression, Stafne bone defect

## Abstract

Asymptomatic lingual depression in the mandible should be evaluated with advanced radiographic modalities such as cone‐beam computed tomography (CBCT) using the software features to achieve correct diagnosis and avoid unnecessary endodontic and/or surgical interventions.

## Graphical abstract

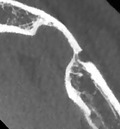

## 1 INTRODUCTION

Mandibular bone depression (MBD) or the Stafne defect is a pseudocyst in the mandible first described in 1942.^1^ It is categorized as a pseudocyst because of its shape, which is in the form of a round cavity in the lingual surface of the mandible simulating the appearance of a cyst. It is not a cavity in bone and is not lined by epithelium.^2^ MBD has a well‐defined margin and is corticated; although a multilocular appearance with ill‐defined borders, full of fat tissue in the posterior molar region has also been reported.^3^

Although the pathogenesis of MBDs is not known, their surgical exploration has shown the presence of a number of different tissues in the defect, including the salivary gland tissue.^4^ MBDs in the posterior mandible have an incidence of 0.10%‐0.48%,^5^ and many of them are likely to remain undetected. The incidence of MBDs is much lower in the premolar area of the mandible, around 0.009%.^2^, ^6^ MBD in the anterior lingual mandibular salivary gland was first reported by Richard and Ziskind in 1957.^7^ Based on a study conducted in 2019, only 62 MBDs in the anterior lingual mandibular salivary gland have been reported in the English‐language literature since 1957.^8^

Unlike posterior MBDs, MBDs in the premolar region are less common and difficult to diagnose due to other similar radiolucent lesions in this region. The aim of this study was to report a new case of MBD in the premolar region focusing on cone‐beam computed tomography (CBCT) analysis.

## 2 CASE REPORT

**Figure 1:**
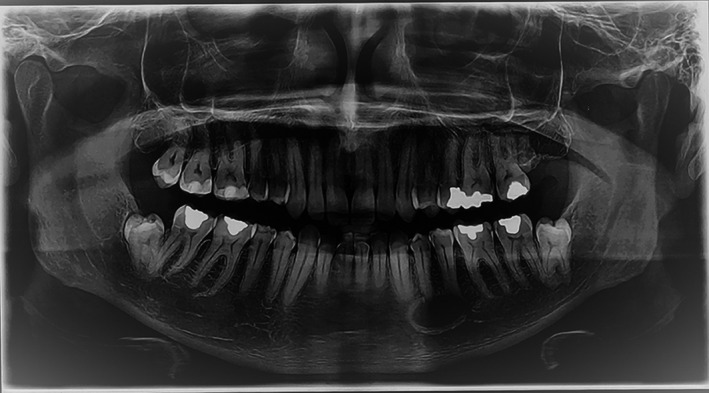
Panoramic view showing a well‐defined radiolucency in the apical region of canine to second premolar teeth

A 40‐year‐old male patient was referred to a private clinic for endodontic treatment of teeth with a periapical lesion in his premolar region of mandible that was discovered during routine radiographic examination. Panoramic radiography revealed a unilocular periapical radiolucency below the apex of the mandibular left canine extending to the second premolar (Figure 1). On the periapical radiograph, the lesion mimicked a radicular cyst; however, the teeth were asymptomatic and the periodontal ligament space and lamina dura were intact. The teeth also gave a positive response to the sensibility tests. The patient had no history of trauma to the jaw. Clinical examination revealed no facial asymmetry and no lymphadenopathy. The most likely diagnosis was a nonodontogenic cyst.

**Figure 2:**
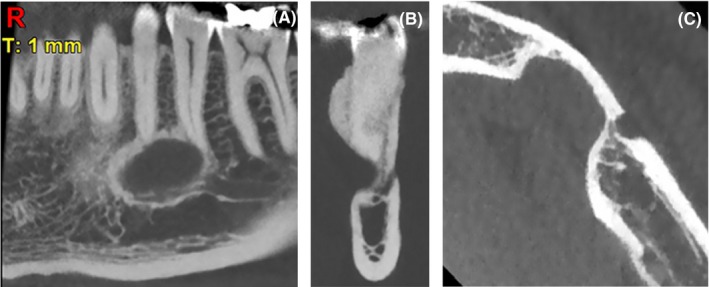
Sagittal CBCT section (A) showing a well‐defined lesion with a cortical border in the periapical region of canine to second premolar teeth. The root apices of the first and second premolars were in contact with the lesion with no sign of resorption. The coronal CBCT plane (B) revealed that the mental foramen was not part of the lesion and was demarcated from the radiolucency with cortical bone. The axial section (C) showing soft tissue invagination into the lingual cortical bone confirming that the mental foramen was not part of the lesion

It was decided to examine the lesion by CBCT to evaluate the relationship of the lesion with its surrounding structures and reach a definite diagnosis. CBCT images were taken by NewTom CBCT system (NewTom VG) with the exposure settings of 110 kVp, 59.62 mA, 4.3 seconds exposure time, 5 × 5 cm field of view, and 14‐bits gray scale. The CBCT scans revealed a well‐defined lingual defect in the canine to second premolar region with no connection to the base of the mandible and a cyst‐like appearance. Evaluation of axial, coronal, and sagittal sections confirmed a lingual wall defect; the buccal wall was intact and there was no expansion. The mental foramen was clearly demarcated from the radiolucency by cortical bone and was not part of the lesion. The root apices of the first and second premolars directly contacted the lesion with no sign of resorption (Figure 2).

**Figure 3:**
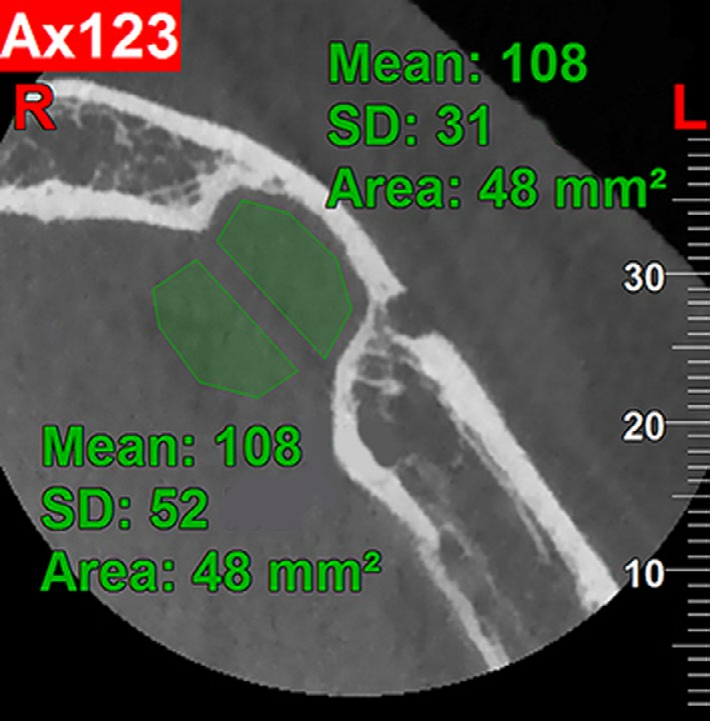
Axial CBCT section: The adjacent lingual soft tissue presented the mean density (gray scale value) similar to that of the lingual depression

For more evaluation of the internal contents of the defect, we used the software's ability to determine the gray scale value. The gray scale value of the main contents of the defect in the middle section was approximately in the range of −78 to 202 with a mean (± standard deviation) of 108 (±31). The mean gray scale value for the adjacent lingual soft tissue was 108 (±52). The lesion was clearly related to the adjacent lingual soft tissue, which could be the anatomical location of sublingual gland with approximately similar gray scale value (Figure 3). Thus, this defect could be classified into the group with range of soft tissues.

Considering the dental and clinical history of the patient and the radiographic features, the defect was diagnosed as a premolar region variant of lingual bone depression and regular follow‐ups were scheduled for the patient.

## 3 DISCUSSION

Mandibular bone depression is found in the mandible, commonly in the posterior part and below the mandibular canal. MBD in the premolar region is rare and unlike the posterior defects, it may be difficult to diagnose due to the absence of guiding anatomical structures. In the premolar area of the mandible, MBDs are often located in the periapical region, sometimes superimposed over the roots and sometimes between or below the roots.^9^ Therefore, they may be misdiagnosed as other radiolucencies or more frequently as cysts (ie, radicular, residual or lateral periodontal cyst, traumatic bone cyst or odontogenic keratocystic), various benign tumors (ameloblastoma), or even bone metastases.^10^, ^11^ Most defects are asymptomatic and nonprogressive. In this case report, this defect was unilocular, in concordance with other studies; however, in the literature, multilocular defects have also been reported, which complicate the diagnosis of MBDs.^12^, ^13^

Mandibular bone depressions are usually discovered during routine radiographic examinations via panoramic radiography. For further assessment, supplemental radiographs are essential. An important clue to the diagnosis is to ensure that the depression is in the lingual surface of the mandible with a thick cortical lining, which could be seen on axial views of computed tomography (CT) and CBCT better than on other radiographic modalities especially for interpretation of MBDs in the premolar region that are often misdiagnosed with periapical lesions.^14^ Segev et al^15^ explained that CT is the next step after panoramic radiography for further investigations and MBD diagnosis; however, they also mentioned that magnetic resonance imaging (MRI) should be considered to identify the contents of the cavity. CBCT does not show soft tissues as well as MRI but CBCT is noninvasive and widely used in dentomaxillofacial radiology in the recent years due to low‐dose radiation. The main advantage of limited volume CBCT is its effective dose, which is lower than CT.^16^ In this case report, further examination was done by CBCT.

Proton‐density, T1‐weighted, and gadolinium‐enhanced T2‐weighted sequences of MRI are especially used for glandular tissues.^17^ Although the etiology of MBDs is not exactly clear, there are two commonly accepted hypotheses in this respect: One of them considers chronic pressure from the glandular tissue on the mandibular lingual cortex to cause lingual resorption while the second hypothesis suggests that it could be due to entrapment of salivary gland tissue during mandibular development.^5^, ^18^ Therefore, detection of salivary gland tissue with MRI can confirm the diagnosis of MBD without surgical biopsy. Consistent with the accepted hypotheses, posterior and premolar area variants of MBDs are related to the hypertrophic lobe of the submandibular and aberrant lobe of the sublingual glands, respectively.^19^, ^20^

In a previous study (2014), CBCT combined with MRI was used as the diagnostic tools. It has been shown that MRI can be a useful diagnostic tool for further evaluation of MBDs suspected on panoramic radiographs.^17^ In the present study, we used the CBCT software's feature to determine the gray scale value and quantitative nature of the internal contents of the MBD.

Gray scale values from CBCT images are affected by the device‐ and scanner‐related parameters, such as voxel size, field of view, and the type of detector.^21^ However, despite the confounding factors, some studies have been able to show a correlation between the Hounsfield units (HUs) of CT and gray scales of CBCT.^22^, ^23^ Kaya et al,^24^ in 2015 examined the bone density around teeth with periapical lesions using CBCT. It should be stated that CBCT units are insufficient for evaluation of soft tissue, because of the inherent scattered radiation and artifacts.^2^

In this study, the gray scale value approximately ranged from −78 to 202, which appears to be due to the presence of fatty tissue in this defect. According to a textbook on salivary glands, the fat content of sublingual glands is less than that of parotid and submandibular glands and therefore its density is higher than that of the other two glands.^25^ In this case report, the mean (± standard deviation) gray scale value of the MBD was approximately 108 (±31), considering the fact that sublingual secretions are predominantly mucinous. This defect can be classified under the category of soft tissues, in concordance with previous reports that examined MBDs with surgery and MRI and found inflamed connective tissue, fatty tissue, striated muscle, and salivary gland tissues in its histopathological analysis.^10^, ^26^, ^27^ However, studies with larger sample size are required to achieve more appropriate conclusions.

## 4 CONCLUSION

Mandibular bone depressions in the premolar region are rare, frequently posing difficulties in diagnosis. In CBCT, its density was approximately similar to that of the adjacent soft tissue, which was the anatomical location of sublingual gland. CBCT is useful in such cases to avoid unnecessary endodontic or surgical interventions.

## CONFLICT OF INTEREST

None declared.

## AUTHOR CONTRIBUTION

SA: selected and managed the patient and contributed to final approval of the manuscript. NA: contributed to manuscript preparation and final approval of the manuscript.
